# Enzymatic Synthesis of Fatty Acid Isoamyl Monoesters from Soybean Oil Deodorizer Distillate: A Renewable and Ecofriendly Base Stock for Lubricant Industries

**DOI:** 10.3390/molecules27092692

**Published:** 2022-04-22

**Authors:** Rafael de Araujo-Silva, Ana Carolina Vieira, Roberto de Campos Giordano, Roberto Fernandez-Lafuente, Paulo Waldir Tardioli

**Affiliations:** 1 Graduate Program in Chemical Engineering (PPGEQ), Laboratory of Enzyme Technologies (LabEnz), Department of Chemical Engineering, Federal University of São Carlos (DEQ/UFSCar), Rod. Washington Luiz, km 235, São Carlos 13565-905, SP, Brazil; rafa.araujo.silva@outlook.com (R.d.A.-S.); ana.acv@live.com (A.C.V.); roberto@ufscar.br (R.d.C.G.); 2 Departamento de Biocatálisis, ICP-CSIC, Campus UAM-CSIC, 28049 Madrid, Spain; 3 Center of Excellence in Bionanoscience Research, External Scientific Advisory Board, King Abdulaziz University, Jeddah 21589, Saudi Arabia

**Keywords:** soybean oil deodorizer distillate, eversa transform, isoamyl fatty esters, biolubricant base stock

## Abstract

In this study, soybean oil deodorizer distillate (SODD), a mixture of free fatty acids and acylglycerides, and isoamyl alcohol were evaluated as substrates in the synthesis of fatty acid isoamyl monoesters catalyzed by Eversa (a liquid formulation of *Thermomyces lanuginosus* lipase). SODD and the products were characterized by the chemical and physical properties of lubricant base stocks. The optimal conditions to produce isoamyl fatty acid esters were determined by response surface methodology (RSM) using rotational central composite design (RCCD, 2^3^ factorial + 6 axial points + 5 replications at the central point); they were 1 mol of fatty acids (based on the SODD saponifiable index) to 2.5 mol isoamyl alcohol, 45 °C, and 6 wt.% enzymes (enzyme mass/SODD mass). The effect of the water content of the reactional medium was also studied, with two conditions of molecular sieve ratio (molecular sieve mass/SODD mass) selected as 39 wt.% (almost anhydrous reaction medium) and 9 wt.%. Ester yields of around 50 wt.% and 70 wt.% were reached after 50 h reaction, respectively. The reaction products containing 43.7 wt.% and 55.2 wt.% FAIE exhibited viscosity indices of 175 and 163.8, pour points of −6 °C and −9 °C, flash points of 178 and 104 °C, and low oxidative stability, respectively. Their properties (mainly very high viscosity indices) make them suitable to be used as base stocks in lubricant formulation industries.

## 1. Introduction

Synthetic base stocks from bio-based sources produced by chemical modifications of natural oils are promising candidates to formulate environmentally friendly lubricants [1,2,3]. Although vegetable oils have some good lubricant properties, their poor oxidative stability and cold temperature properties (e.g., high pour point) must be corrected [1,2,4]. Several routes can be adopted to chemically (or enzymatically) modified natural oils, such as epoxidation, hydrogenation, estolide formation (branched esters formed by linking the carboxyl group of one fatty acid to a site of unsaturation of another fatty acid), and transesterification/esterification or to obtain straight or branched-chain monoesters, diesters, triesters, and polyol esters [1,2,3,4,5,6,7,8]. Organic esters can solve some of the oil limitations [2,4,9]. According to American Petroleum Institute (API), the esters are utilized as standalone base stocks or as blend components [9].

Ester-based base stocks for lubricant formulations can be synthesized by transesterification of oils or fats or esterification of free fatty acids (FFAs), with monohydroxy or polyhydroxy alcohols, catalyzed by homogenous or heterogeneous inorganic catalysts or enzymes [2,6,7,10,11,12]. The high pour points, low viscosities, and oxidation stabilities make them useful in metalworking [13]. To be considered as biolubricant, the ester-based blend must be biodegradable [14] and present low human and environmental toxicity, besides obviously performing its desired function, such as decreasing friction and heat, protecting against corrosion and wear, or transmitting energy [2,4,15]. Monoester-based biolubricants are frequently applied as hydraulic fluid or lubricant additives; on the other hand, polyolester-based biolubricants also have possibilities to be used in transport, food industries, and greases [16].

Currently, biolubricants are a small fraction (about 2%) of the global lubricant market [18], but this small market share has a solid potential to grow [17,18,19].

Despite the environmental benefits of bio-based lubricants compared with mineral oils, the use of edible vegetable oils as raw material in the biolubricant manufacture is of particular concern [2]. For example, soybean, as well as its oil, is widely used in food and feed industries [20]. Using soybean oil as raw material in energy industries to produce biodiesel could impact its availability and price, generating social problems [21]. To circumvent this problem, fatty acid sources from non-food biomass, wastes, or low-value byproducts could be viable options [8,19,22,23]. 

Soybean oil deodorizer distillate (SODD) is a byproduct of the edible soybean oil refinement process, rich in FFA and acylglycerols (17 to 47 wt.% and 9 to ~70 wt.%, respectively) and with considerable amounts of tocopherols (1.8 to 20 wt.%), a natural antioxidant [24,25,26,27]. Its use as raw material for biodiesel production has been widely reported in the scientific literature [27,28,29,30,31,32,33,34,35,36,37], but its exploitation to produce ester base stocks for the lubricant market is scarce, and even less in terms of enzymatic routes [38]. Lately, Fernandes et al. (2021) investigated the esterification of FFA obtained from acidulation of soapstock of the soybean fatty acid distillate with trimethylolpropane (TMP) and neopentyl glycol (NPG) to produce biolubricant base stocks catalyzed by *Candida rugosa* lipase (CRL)s [38]. 

Linear or non-linear monohydroxy alcohols (e.g., methanol and 2-ethylhexanol) are commonly used to produce monoester base stocks, depending on the tribological properties required for the desired application [13,16]. Fusel oil, a byproduct of the bioethanol distillation [39] could be a source of alcohols to be applied in perfume fixers, essences, or paints [40], but in many instances, they usually are burned in the plant boiler or incorporated in the ethanol combustive. Fusel oil has low costs [41] and a high content of isoamyl alcohol. This has been utilized in the synthesis of esters using palm kernel oil (constituted by high amounts of lauric acid) [42] and oleic acid [43] as acyl donors with high conversions but low kinematic viscosities at 40 °C.

Despite being cheaper, chemical catalysts are less selective (producing many by-products) and need harsher reactional conditions than enzymatic catalysts [44]. Many vegetable oils and esters may be damaged under these conditions [5,45], making lipases a good alternative for this application. Lipases are non-toxic and environmentally friendly biocatalysts, capable of promoting both esterification and transesterification reactions between FFAs and oils or fats as acyl donors, respectively, and alcohols as acyl acceptors under mild conditions [27,46,47,48,49,50]. Lipases usually have high organic solvent stability, wide substrate specificity, and high enantioselectivity, and do not need cofactors [46,47,51]. Among the commercially available lipase, Eversa Transform 2.0 (a liquid formulation of an industrial variant of the lipase from *Thermomyces lanuginosus*) has been launched as a biocatalyst suitable for the synthesis of biodiesel in its liquid form, although some authors have shown the possibility of improving this feature after immobilization [27,50,52,53,54]. This enzyme has shown reaction rates comparable to transesterification of glycerides and esterification of FFA with methanol to produce fatty acid methyl ester (FAME) [55,56]. Eversa Transform 2.0 (ETL 2.0) is more thermostable and has higher specific activity than their previous version [55,56,57], and is also more stable than the original lipase from *Thermomyces lanuginosus* (TLL) [58]. Despite its several advantages in the use of immobilized lipases [59,60], in the case of simultaneous esterification/transesterification of free-fatty-acid-rich feedstocks, the water generated throughout the esterification reaction and accumulated within the biocatalyst particles could be a drawback [61]. Although there are alternatives to circumvent this problem, such as the use of ultrasound [62,63,64] and highly hydrophobic supports [65,66,67,68,69,70], the use of lipase in its free form is an appealing alternative because the water problem in the bulk can be easily circumvented using molecular sieves [27,71,72]. Liquid Eversa formulations are highly active (low concentrations of different Eversa versions were required to achieve more than 95% of FAME yield in 16 h reaction under mild temperatures [55,56,73]), operationally stable, and cheaper than other commercial lipases [55,56,57].

In this context, this study aimed to evaluate the production of fatty acid isoamyl esters (FAIE) from crude SODD, catalyzed by liquid Eversa formulation. Considering the higher value of biolubricant compared with biodiesel, if the use of Eversa is feasible in biodiesel production, we can expect that it can be in biolubricant base stock production. A design of experiments was performed to select some independent variables (temperature, acyl donor (as saponifiable matter)/alcohol molar ratio, and percentage of enzyme mass regarding the mass of crude SODD) capable of enhancing the FAIE through esterification/transesterification of SODD with isoamyl alcohol. The control of water concentration in the initial reaction medium and throughout the reaction was also investigated. A small amount of water in the reaction medium is required to maintain the enzyme 3D structure, thus preserving its active site polarity and protein stability adequate to the catalysis. However, a large amount of water can negatively affect the esterification equilibrium toward the hydrolysis reaction of the newly formed ester [74]. For ETL-2.0, in particular, Novozymes [57] recommends a reactional medium with 2% water for the best performance of enzyme in the synthesis of biodiesel using methanol as an acyl acceptor. The use of a molecular sieve (MS) in the reaction medium permits the capture of the excess water formed during the esterification reaction, increasing the esterification yield [63,75,76]. Finally, the products (isoamyl esters) obtained under the optimal conditions were characterized as to some of their physicochemical properties (viscosities at 40 °C and 100 °C, viscosity indices, pour points, flash points, oxidative stability, and copper corrosiveness).

## 2. Results

### 2.1. Physicochemical Characterization of Soybean Oil Deodorizer Distillate (SODD)

The saponifiable composition and physicochemical proprieties of crude SODD are shown in Table 1. The crude SODD is saponifiable-rich biomass (>90 wt.%) composed mainly of triglycerides (~70 wt.%) and free fatty acids (18.5 wt.%).

In general, a good lubricant base stock should have high viscosity index, high flash point, low pour point, good corrosivity resistance, and high oxidation stability [4]. The crude SODD exhibited physicochemical properties suitable to be used as biolubricant base stock, such as a very high viscosity index, ~192 (the higher the viscosity index, the smaller the changes in viscosity over a broader temperature range) and high flash point, at 210 °C (desirable to warrant secure operation at high temperatures) [2,5], as well as being non-corrosive to copper (Table 1). Although the crude SODD already has interesting properties as lubricant bases, its free acidity (~18 wt.%) and pour point (−3 °C) are high, and its oxidative stability is low, but the esterification/transesterification of their saponifiable matter could improve these parameters and increase the range of applications in lubricant formulation industry.

### 2.2. Rotational Central Composite Design (RCCD) and Analysis by Response Surface Methodology (RSM)

From the results shown in Table 2, the regression coefficients of a third-order model of the type $y=a_{0}+b_{i}x_{j}+c_{i}x_{j}x_{k}+d_{i}x_{j}{}^{2}+e_{i}x_{j}{}^{3}\;$ were calculated, and a mathematical model using Equation (1) was built for the response FAIE yield only using significant terms (*p*-value < 0.05). ANOVA was used to evaluate the accuracy of the fitted model (Table 3). The FAIE yields (Table 2) varied from 1.22 wt.% to 48.62 wt.%, and the R^2^ and calculated F-value (Table 3) were suitable to obtain a third-order model using Equation (1), allowing the evaluation of the FAIE yield as a function of R_isoamyl_ (*x*_1_) and m_enz_ (*x*_3_), within the range studied. The third-order model was chosen based on the literature recommendation when a second-order model is not capable of accurately describing the experimental results [77].

The mathematical model was built using the coded variables with statistically significant parameters (at a 5% significance level; the temperature was not statistically significant in the evaluated range), according to the measured values for the response, defined by Equation (1).

(1) $$Y\left[\mathrm{wt}.\%\right]=41.21+3.46x_{1}+6.74x_{1}x_{3}-4.21x_{1}^{2}-16.12x_{3}^{2}+10.30x_{3}^{3}$$

where $x_{1}$ and $x_{3}$ are the independent variables coded for R_isoamyl_ and m_enz_, respectively.

**Figure 1:**
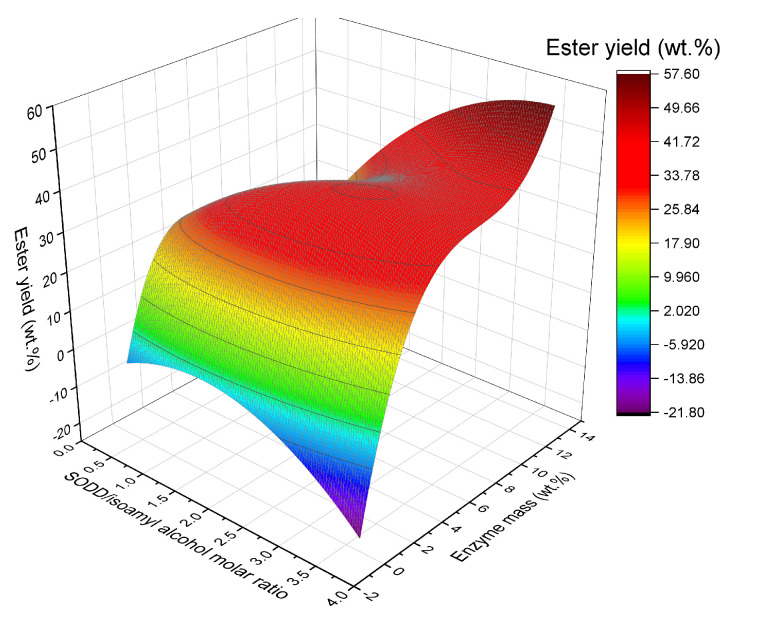
Response surface showing the effects of independent variables on the yield of fatty acid isoamyl ester constructed from model Equation (1).

Equation (1) was used to generate the response surface for the FAIE yield (Figure 1), which showed a fast growth of Y between the range of 0 wt.% and 6 wt.% of m_enz_. From 6 wt.% of m_enz_ upward, the Y growth was less pronounced. Aiming to combine ester productivity and enzyme consumption, it was established that the first turning point observed in the response surface would be the best operating condition for R_isoamyl_ and m_enz_, i.e., 1:2.5 wt.% and 6.0 wt.%, respectively. The reaction temperature was set at 45 °C, the level zero condition for this variable of the experimental design, for the further assays, due to this value being reported as a secure operational temperature for ETL 2.0 [55,57].

To validate the model, an experimental assay (in triplicate) was carried out under the designed optimal conditions (R_isoamyl_ of 1:2.5, m_enz_ of 6.0 wt.%, 45 °C, and stirring at 250 rpm in a shaker incubator) gave a reaction yield of 35.0 ± 2.5 wt.%, while the model Equation (1) predicted a reaction yield of 41.9 ± 6.8 wt.% (a relative error between experimental and predicted value less than 20%). This result shows that the model could satisfactorily predict the real behavior of this complex reaction system.

**Figure 2:**
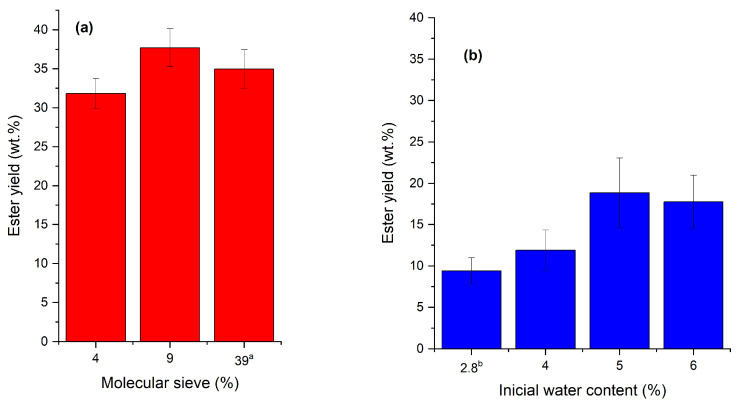
Reaction yields (*Y*, wt.%) of esterification/transesterification of SODD with isoamyl alcohol (molar ratio of 1:2.5, 6.0 wt.% of ETL 2.0, 45 °C, and stirring at 250 rpm in a shaker incubator for 12 h) as a function of water content in the reaction medium: (**a**) without added water and using molecular sieve (MS); (**b**) without MS and adding water at the beginning of the reaction. ^a^ Almost anhydrous condition throughout the reaction. ^b^ Only water content already contained in the reagents and enzyme (without molecular sieve or addition of water).

As previously discussed in the Section 1, the content of water in the reaction medium is an important parameter to be controlled aiming to retain the enzyme activity and prevent the hydrolysis of the formed ester. Based on the adsorption capacity of MS informed by the manufacturer (0.20 g of water per gram of MS), the addition of 4 wt.% and 9 wt.% of MS in the reaction medium was enough to reach around 2 wt.% and 1 wt.% water at the beginning of the reaction, respectively. The addition of 39 wt.% MS should be enough to guarantee an almost anhydrous medium throughout the reaction. Therefore, the presence of 4, 9, and 39 wt.% MS (MS mass/SODD mass), and the addition of additional water to the reaction medium (without MS), ranging from 2.8 wt.% (percentage of water already contained in the reagents and enzyme) to 6 wt.%, were evaluated to determine the influence of water on the reaction yield. Figure 2 shows the reaction yields (*Y*) with (Figure 2a) and without (Figure 2b) MS.

The ester synthesis had better performance in a water-controlled medium (Figure 2a), resulting in an ester yield almost twofold higher than that in a medium where the water content was only adjusted at the beginning of the reaction (Figure 2b). The water control during the reaction could favor the displacement of the equilibrium of the reaction towards the product while maintaining a water amount necessary to the enzyme catalysis [61]. Differently, Remonatto et al. [56] reported better performance of Eversa in the esterification/transesterification of oleic acid/soybean oil (mass ratio of 1:1) with methanol (97% conversion after 16 h reaction) with 2.5% added water. In this case, the addition of water was necessary because methanol can drastically inactivate the enzyme due to the removal of the enzyme water layer required for the catalysis [78,79].

### 2.3. Reaction Course under Optimal Conditions

**Figure 3:**
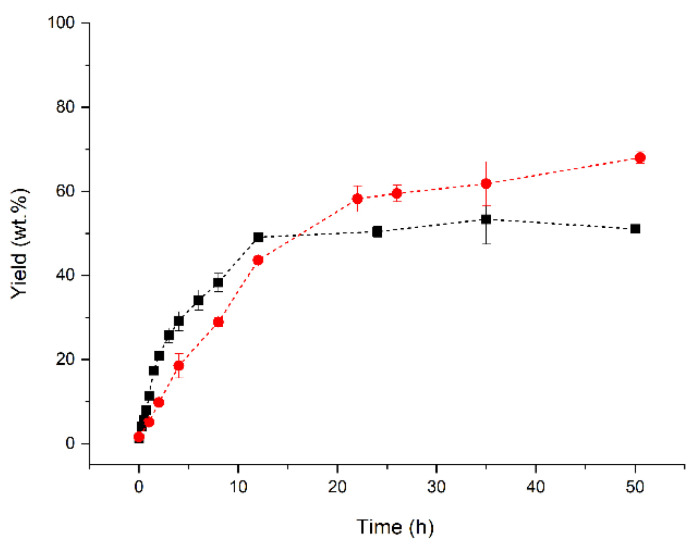
Profiles of ester mass yield vs. reaction time for esterification/transesterification of SODD with isoamyl alcohol in presence of two MS concentration (9 wt% (●) and 39 wt% (■)). Reaction conditions: SODD_Sap_/isoamyl alcohol molar ratio of 1:2.5, 6.0 wt.% Eversa Transform 2.0 (enzyme mass/SODD mass) and 45 °C.

Under optimal reaction conditions (SODD_Sap_:isoamyl alcohol molar ratio of 1:2.5, 45 °C and 6.0 wt.% ETL 2.0 (enzyme mass/SODD mass), the esterification/transesterification reaction of SODD with isoamyl alcohol was carried out for two distinct MS conditions—9 wt.% and 39 wt.%—monitoring the reaction yield (Y) with the time reaction. Figure 3 shows a better global evolution of the reaction using 9 wt.% MS, reaching almost 70 wt.% yield (ester mass/product mass) after 50.5 h reaction, but when using 39 wt.% MS, the reaction stopped after 12 h reaction, at around 50 wt.% yield. Although the lack of water using 39 wt.% MS favored the reaction for the first 12 h, this result suggests that, at this time span, the lack of water probably inactivated the enzyme. Moreover, it should be considered that this is a solvent-free reaction. This means that the composition of the medium is changing along the process, initially, the medium properties are defined by those of the substrate mixture, while at the end, the medium features are influenced by the isoamyl ester [80]. These dynamic changes may affect the role of water activity in the performance of the enzyme. Therefore, a small amount of water resulting from the use of 9 wt.% MS could be essential to preserve catalyst activity when there is a high ester concentration in the medium and, consequently, keeping going on the reaction [81,82], even though it may not be so relevant in the initial steps.

### 2.4. Chemical Composition and Some Physicochemical Properties of the Products

SODD and reaction products with 12 and 22 h reaction times (product 1 containing 43.7 wt.% FAIE and product 2 containing 55.3 wt.% FAIE, respectively) were characterized regarding their physicochemical properties and chemical compositions (Table 4).

As shown in Table 1 and discussed in Section 2.1, crude SODD has some interesting properties to be used as lubricant base stocks (results are listed again in Table 4 only for comparison purposes). Probably, due to the high triglyceride amount (more than 70 wt.%), SODD exhibited some physicochemical properties similar to those of refined soybean oil [23]. However, some properties shown in Table 1 were improved after the transesterification/esterification reaction. In general, Table 4 shows that the higher the content of isoamyl esters in the reaction products, the smaller the viscosities at 40 °C and 100 °C, the viscosity index, relative density, and the pour and flash points, but the higher the oxidative stability. In general, based on the physicochemical properties (viscosities at 40 and 100 °C, viscosity index, and flash point), both synthesized lubricant base stocks could serve to formulate hydraulic fluids [9,16,83,84].

A high viscosity index is an essential characteristic of a good lubricant base stock since it is an indication that the lubricant can be used over a wide range of temperatures by maintaining the thickness of the oil film [4]. SODD and products 1 and 2 had viscosity index similar to those of commercial synthetic lubricant base stocks according to the BASF Esters-Base-Stocks Selection Guide for Lubricants and Metalworking Fluids [16]. A high viscosity index (>160) could be associated with the polyunsaturated fatty acids chains of the soybean oil because it is reported that branched biolubricant (in the alcohol or in the carboxylic acid) favors a lower viscosity index [2].

The flash point is greatly impacted by the ester amount in the product but is high enough to ensure safe operations at moderate temperatures. The flash point of product 2 was lower than that of product 1, which could be related to the remaining amount of isoamyl alcohol (~1 wt.%, determined by gas chromatography) after drying. On the other hand, the pour point decreased until 6 °C, with the increase of the concentration (in wt.%) of isoamyl esters in the products, probably because the branched groups of isoamyl alcohol did hamper the acyl chains to come close for easy stacking because of steric interactions, thus inhibiting crystallization, resulting in a lower pour point [1]. However, all products had poor pour points to work at low temperatures [4], as cloudy conditions, precipitates, and solidification could not be avoided upon long-term exposure to cold temperatures, resulting in poor flow and pumpability [4]. Meanwhile, this problem, as well as some others, can be easily solved in the lubricant formulation by adding certain additives (e.g., pour point depressants, antioxidants, and viscosity modifiers) and diluents (e.g., 2-ethylhexyl oleate, isobutyl oleate, dibutyl adipate, di-isodecyl adipate, high oleic vegetable oils) or functional fluids in order to fulfill the requirements of a specific application [1,2,4,5]. For example, the addition of a 1% pour point depressant (e.g., Lubrizol TM 7670 made from sunflower and mineral oils) reduced the pour point of soybean-oil-based lubricant from −9 °C to −45 °C [4].

The oxidative stability was not expected to improve greatly because the unsaturated nature of fatty acids in soybean oil makes them prone to rapid oxidation (mainly linolenic and linoleic acids) [4,85] and, in turn, the SODD and their isoamyl esters. The oxidative stability of product 2 increased by 10 min, compared with SODD, most likely due to the elimination of the β-CH group of the acylglycerol structure, highly susceptible to thermal instability [4]. As discussed above, this parameter can be improved by adding antioxidants in the lubricant formulation depending on the desired application. The presence of tocopherols, a natural antioxidant, is reported to improve the oxidative stability of unrefined vegetable oils, compared with refined ones [86,87]. In fact, naturally occurring antioxidants such as tocopherol, l-ascorbic acid, esters of gallic acid, citric acid derivatives, or EDTA derivatives have been reported to serve as synthetic metal scavengers and provide viable alternatives to the currently used toxic antioxidants [5]. However, the low content of tocopherols in the SODD [27], and thus in the reaction products, was not enough and/or inefficient to reach high oxidative stability at harsh assay conditions (measured by RPVOT method in a sealed cell pressurized with oxygen and submitted to a temperature of 150 °C). Thus, the improvement of this parameter requires the use of suitable antioxidants or even a chemical modification of acyl moieties double bonds of the isoamyl esters, depending on the desired applications [5].

The acid value of crude SODD was greatly reduced with the reaction, but when using only 9 wt.% MS, this decrease was not enough. The presence of some water could permit the glycerides hydrolysis, probably impacting the product viscosity. On the other hand, the presence of water avoided lipase inactivation, allowing a reaction yield to be reached that is higher than that achieved using 39 wt.% molecular sieves. However, FFAs and monoglycerides (higher in product 2 than product 1), such as glycerol monooleate, are known as friction modifiers, with high tribological appeal [88,89].

Synthetic esters-based lubricants still display several limitations to compete with mineral lubricants, since chemical modification raises the price of the lubricant, slightly increases the volatility and toxicity, and diminishes the friction tolerance, and the esters do not work well with mineral oils in comparison to unmodified vegetable oils [90]. Resources regarding the improvement of some properties must continue, mainly regarding the improvement of oxidative stability and pour point. Besides the use of additives mentioned above, modification of unsaturated acyl chains by chemical processes (epoxidation followed by ring opening) is a promising alternative to improve oxidative stability [2,4,6,8]. On the other hand, the goals for environmental protection are essential to change this scenario. Beyond that, vegetable oil products are ideally suited for applications such as lubrication of sawmill blades or chain drives, whereby the lubricant is used on a single-use basis [4]. However, to reach a final decision, a complete tribological study (study of friction, lubrication, and wear) must be carried out.

## 3. Materials and Methods

### 3.1. Materials

Eversa Transform 2.0 (ETL 2.0, 1.2 g/mL density, 25 mg/mL protein, and 5425 Tributyrin Unit (TBU)/mg specific activity) and methyl heptadecanoate were purchased from Sigma-Aldrich (St. Louis, MO, USA), molecular sieve (MS) rods (1/16 in, 3 Å pore size, and water capacity of 0.20–0.23 g of water per gram of MS) were supplied by Fluka (Charlotte, NC, USA), isoamyl alcohol was from Êxodo Científica (Sumaré, SP, Brazil), and soybean oil deodorizer distillate (SODD) was kindly donated by COCAMAR (Maringá, PR, Brazil). All other chemicals and solvents were of analytical or HPLC grade, and they were used as received.

### 3.2. Rotational Central Composite Design (RCCD)

The assays were carried out in sealed bottles with MS rods to provide a reaction medium near anhydrous conditions (32.7–56.7 wt.%, depending on the initial water content). The bottles were incubated in a shaker incubator (Marconi, MA832/H) at a 250 rpm stirring speed for 12 h. In the end, the reaction media were washed with boiling distilled water in a volume ratio of 1:1 (water:SODD), centrifuged at 10,400 rcf (9645 rpm) for 10 min at 25 °C, and the polar phase was discarded (this washing procedure was repeated 3 times). The samples were dried on a lab stove at 50-60 °C for approximately 24 h, and the reaction mass yields were calculated from the FID–GC results.

The independent variables SODD saponifiable (SODD_Sap_)/isoamyl alcohol molar ratio (R_isoamyl_, 1:0.318 to 1:3.682), reaction temperature (T, 19.8 to 70.2 °C), and enzyme mass in the reactor per SODD mass (m_enz_, 1 to 13.1%) were chosen to carry out the RCCD (2^3^ factorial with 6 axial points and with 5 repetitions at the central point) totaling 19 trials (Table 5) to evaluate the effects of these variables on the FAIE yields. Data were analyzed by Statistic Software, and a third-order model was obtained and evaluated statistically by analysis of variance (ANOVA).

After model validation and using the reaction conditions that yielded the highest ester yield, the water initial content of the reaction medium ranged from 1 wt.% to 6 wt.% (based on the MS capability to adsorb 0.20 g of water per gram of MS), adding the MS to adjust the initial water content in the reaction medium) or adding water (without MS). The reactions were performed in sealed bottles (with plugs and caps) incubated in a shaker incubator (Marconi, MA832/H) at 250 rpm stirring speed for 12 h. In the end, the reaction media were treated as described above to quantify the FAIE yields by gas chromatography.

### 3.3. Reaction Courses

The reaction courses were studied under the operational conditions previously selected (Section 3.2). The reaction was conducted in a jacketed reactor (80 × 35 mm, H × Di) with mechanical stirring with a straight paddle impeller (25 × 7 mm, L × H). Samples were withdrawn at regular time spans, washed with boiling distilled water, and dried, as described in Section 3.2, before being analyzed by gas chromatography (Section 3.4).

### 3.4. Chromatographic Analysis

#### 3.4.1. Ester Analysis

FAIE were quantified by gas chromatography according to EN 14103 standard [91], with modifications. A mass of 50 mg of dry reaction sample was weighted in a vial and solubilized in 1.0 mL of a methyl heptadecanoate solution (1.0 mg/mL in heptane) as the internal standard. The analyses were carried out in a 7890-A Agilent chromatograph (Agilent Technologies, Santa Clara, CA, USA) using an Rtx-Wax capillary column (30 m × 0.25 mm × 0.25 μm, Restek Co., Bellefonte, PA, USA), injector temperature of 250 °C, and helium as carrier gas (19.91 psi, flow rate of 54 mL/min and split rate of 50:1). A volume of 1 μL of the sample was injected into the device, with the oven and FID detector set at 210 °C and 250 °C, respectively, and hydrogen, synthetic air, and nitrogen flow rates of 30, 400, and 25 mL/min, respectively.

The reaction yield (*Y*, in wt.%) for each sample was calculated by Equation (2),

(2) $$Y\;\left[\mathrm{wt}.\%\right]=100\cdot \frac{\sum A_{i}}{A_{standard}}\cdot \frac{C_{standard}\cdot V_{standard}}{m_{sample}}$$

where $A_{i}$ is the sum of chromatography areas of the chromatogram peaks at the retention time of isoamyl esters, $A_{standard}$ is the area of methyl heptadecanoate peak, $C_{standard}$ is the mass concentration of methyl heptadecanoate solution in heptane, $V_{standard}$ is the volume of methyl heptadecanoate solution (1 mL, in heptane), and $m_{sample}$ is the mass of reaction sample (~50 mg).

#### 3.4.2. Analysis of Acylglycerides and Glycerin

Total monoglycerides, diglycerides, triglycerides, and glycerin were quantified by gas chromatography according to the ASTM D6584 standard [92], with modifications. The analyses were carried out in a 7890-A Agilent chromatograph (Agilent Technologies, Santa Clara, CA, USA)) using a Select Biodiesel column (glycerides, UM + 2 m RG, 15 m × 0.32 mm × 0.1 µm, Agilent Technologies, Santa Clara, CA, USA), injector with a pressure of 7.52 psi, and 3 mL/min helium (carrier gas) flow rate. A volume of 1 μL of the sample was injected into the column, and the analysis was run under the following oven temperature ramp, with an initial temperature at 50 °C, followed by heating rates of 15, 7, and 30 °C/min to reach 180, 230 and 380 °C, respectively; the FID detector was set at 300 °C with hydrogen, synthetic air, and nitrogen flow rates of 30, 400 and 25 mL/min, respectively.

### 3.5. Chemical and Physicochemical Characterizations of the Reaction Products

Water content was quantified according to the Karl Fisher method [93], and acid value and saponification value were determined according to AOCS Cd 3d-63 [94] and Cd 3-25 [95] standards, respectively. Viscosities at 40 and 100 °C, viscosity index (VI), density, corrosiveness to copper, pour point, flash point, and oxidation stability (by rotatory pressured vessel oxidation test-RPVOT) were determined by ASTM standards D445, D2270, D1298, D130, D97, D93, and D2272, respectively, at the laboratories Lubrin Tribological Analyses (São Paulo, SP, Brazil) and SENAI (São Paulo, SP, Brazil). Samples were washed three times with boiling point water, and the organic phase was dried in an oven at 60 °C until constant weight.

## 4. Conclusions

The soybean oil distillate deodorizer (SODD) showed to be a promising source of acyl donor to produce isoamyl esters via enzymatic catalysis. The partial conversion of the fatty acids (in form of free fatty acids or glycerides) from the SODD produced base stocks with some interesting lubricant properties, mainly high viscosity index (>160). The synthesized lubricant base stocks have suitable properties to be used as hydraulic fluid or as biolubricant additive. Nevertheless, for use in other specific applications, its physicochemical properties must be adjusted.

## Figures and Tables

**Table 1:** Saponifiable composition and physicochemical properties of soybean oil deodorizer distillate (SODD) from Cocamar (Maringá, PR, Brazil), collected in June 2019.

Parameter	Value
Saponification value (mg_KOH_/g) Saponification value (wt.%)	186.75 ± 4.25 93.84 ± 2.13
Acid value (mg_KOH_/g) Acid value (wt.%)	36.85 ± 0.07 18.52 ± 0.03
Moisture (wt.%)	0.18
Relative density	0.921
Viscosity at 40 °C (mm^2^/s)	33.5
Viscosity at 100 °C (mm^2^/s)	7.3
Viscosity index	191.6
Pour point (°C)	−3.0
Flash point (°C)	210
Oxidation stability (min)	43
Corrosiveness to copper	1A ^(a)^
Free glycerol (wt.%)	0.003
Monoglycerides (wt.%)	1.38
Diglycerides (wt.%)	4.39
Triglycerides (wt.%)	69.5

^(a)^ The rating of 1A is given for appearance of freshly polished copper coupons with slight discoloration but barely noticeable.

**Table 2:** RCCD matrix for esterification/transesterification of SODD with isoamyl alcohol catalyzed by Eversa Transform 2.0 in almost anhydrous media (incubated in shaker at 250 rpm stirring for 12 h reaction) with experimental results for fatty acid isoamyl ester yield (*Y*).

Run	SODD_Sap_: Isoamyl Alcohol Molar Ratio (R_isoamyl_, $x_{1}$)	Temperature (^°^C) (T, $x_{2}$)	Enzyme Mass (wt.%) (m_enz_, $x_{3}$)	Reaction Yield (wt.%) (Y)
1	1:1 (−1)	30.0 (−1)	1.0 (−1)	14.63
2	1:1 (−1)	30.0 (−1)	10.0 (+1)	20.00
3	1:1 (−1)	60.0 (+1)	1.0 (−1)	8.71
4	1:1 (−1)	60.0 (+1)	10.0 (+1)	14.81
5	1:3 (+1)	30.0 (−1)	1.0 (−1)	11.79
6	1:3 (+1)	30.0 (−1)	10.0 (+1)	33.76
7	1:3 (+1)	60.0 (+1)	1.0 (−1)	4.56
8	1:3 (+1)	60.0 (+1)	10.0 (+1)	47.96
9	1:2 (0)	45.0 (0)	5.5 (0)	39.79
10	1:2 (0)	45.0 (0)	5.5 (0)	41.45
11	1:2 (0)	45.0 (0)	5.5 (0)	43.29
12	1:2 (0)	45.0 (0)	5.5 (0)	42.83
13	1:2 (0)	45.0 (0)	5.5 (0)	48.62
14	1:0.318 (−1.68)	45.0 (0)	5.5 (0)	29.09
15	1:2 (0)	19.8 (−1.68)	5.5 (0)	38.74
16	1:2 (0)	45.0 (0)	0 (−1.22)	1.22
17	1:3.682 (+1.68)	4)5.0 (0)	5.5 (0)	33.33
18	1:2 (0)	70.2 (+1.68)	5.5 (0)	35.57
19	1:2 (0)	45.0 (0)	13.1 (+1.68)	46.92

**Table 3:** Analysis of variance (ANOVA) for the experimental design of the esterification/transesterification of SODD with isoamyl alcohol catalyzed by Eversa Transform 2.0.

Source of Variation	SS ^a^	DF ^b^	MS ^c^	F_calculated_	F_tabulated_
Regression	4129.56	5	825.91	31.09	3.02
Residue	345.37	13	26.57		
Lack of fit	86.04	3	28.68	1.11	3.71
Pure error	259.32	10	25.93		
Total	4474.92	18	248.61		

^a^ Sum of squares; ^b^ Degree of freedom; ^c^ Mean squares. R^2^ = 92.28% at 5% significance level.

**Table 4:** Physicochemical properties and chemical composition of the SODD and the esterification/transesterification reaction products containing different content of fatty acid isoamyl esters (FAIE).

Parameters	SODD	Product 1 ^a^	Product 2 ^b^	Unity	Standard
FAIE content	0	43.7	55.3	wt.%	
Viscosity at 40 °C	33.5	20.5	13.5	cSt	ASTM D445
Viscosity at 100 °C	7.3	4.9	3.6	cSt	ASTM D445
Viscosity index	191.6	175.0	163.8	-	ASTM 2270
Relative density	0.921	0.911	0.899	-	ASTM D1298
Pour point	−3.0	−6.0	−9.0	°C	ASTM D97
Flash point	210	178	104	°C	ASTM D93
Oxidative stability	43	37	53	min	ASTM D2272
Corrosiveness to copper ^c^	1A	-	1B	-	ASTM D130
Saponification value	186.75 ± 4.25	153.3 ± 11.4	152.2 ± 0.46	mg_KOH_/g	AOCS Cd 3-25
Acid values	36.85 ± 0.07	9.78 ± 0.04	15.45 ± 0.08	mg_KOH_/g	AOCS Cd 3d-63
Free glycerol	0.003	0.25	0.50	wt.%	ASTM D6584
Monoglycerides	1.38	4.36	7.96	wt.%	ASTM D6584
Diglycerides	4.39	9.04	8.38	wt.%	ASTM D6584
Triglycerides	69.5	3.05	1.40	wt.%	ASTM D6584

^a^ Product 1 was synthesized in presence of 39 wt.% molecular sieves for 12 h reaction, and ^b^ Product 2 was synthesized in presence of 9 wt.% molecular sieves for 22 h reaction, both using 1:2.5 (mol:mol) SODD: isoamyl alcohol, 6.0 wt.% (m_enzyme_/m_SODD_) Eversa Transform 2.0 and 45 °C; ^c^ The rating of 1A is given for appearance of freshly polished copper coupons with slight discoloration but barely noticeable; 1B indicates slight tarnish, and the ratings proceed further down the scale as corrosion staining of the test coupon increases, with 4C being the worst, typically appearing as severely corroded, blackened, and pitted coupon.

**Table 5:** Values of independent variables used in the RCCD for enzymatic synthesis of fatty acid isoamyl esters for 12 h reaction.

Factors	−1.68	−1	0	+1	+1.68
SODD_Sap_: isoamyl alcohol molar Ratio (R_isoamyl_) ( $x_{1}$)	1:0.318	1:1	1:2	1:3	1:3.682
Temperature (^°^C) (T) ($x_{2}$)	19.8	30	45	60	70.2
Enzyme mass/SODD mass, wt.% (m_enz_) ($x_{3}$)	0	1	5.5	10	13.1

## Data Availability

The data presented in this study are available on request from the corresponding author.

## Sample Availability

Samples of the compounds are not available from the authors.
